# The interplay between academic performance, emotional intelligence, and self-concept as predictors of violent behavior in higher education: a multi-group structural equation modeling

**DOI:** 10.3389/fpsyg.2023.1124712

**Published:** 2023-05-24

**Authors:** José Luis Ubago-Jiménez, Silvia Corral-Robles, José Luis Ortega-Martín, Eduardo Melguizo-Ibáñez

**Affiliations:** ^1^Department of Didactics of Musical, Artistic and Corporal Expression, Faculty of Education, University of Granada, Granada, Spain; ^2^Department of Languages and Literature Teaching, Faculty of Education Sciences, University of Granada, Granada, Spain

**Keywords:** violent behavior, academic performance, emotional intelligence, self-concept, university students

## Abstract

Higher education is a focus of increasing violent behavior. The evidence suggests an obsession to achieve the best academic performance in order to access working life. This research aims to develop an explanatory model of violent behavior and its relationship with self-concept and emotional intelligence according to in relation to their academic performance. A sample of 932 Spanish undergraduate students participated in the multi-group structural equation modeling. Findings revealed that students who have a higher academic performance have problems to control and regulate their emotions, showing signs of direct and indirect violence. Moreover, it was found that that emotional intelligence and self-concept have a direct influence on episodes of violent behavior, with academic performance being a key component affecting each variable. The present study provides some implications and suggests some avenues for future research.

## Introduction

Violent behaviors in higher education are a focus of international research (Itani et al., 2017; Dobarro et al., 2018; Martínez-Martínez et al., 2018; Esquivel-Santovena et al., 2020; Alsawalqa, 2021). Versatility and adaptability of violence in its physical, psychological or social forms is a challenge to the educational community in general. Recently, it has been possible to see how higher education institutions have deteriorated in terms of coexistence, which has a negative impact on academic performance, engagement, and physical as well as psychological health, even causing students drop out of their studies (Bernardo et al., 2020; MacCann et al., 2020; Martínez-Monteagudo et al., 2020).

In recent years, violent behaviors have been adapted to new human interaction methodologies. Cyberbullying is one of the most widespread consequence in higher education (Watts et al., 2017; Alsawalqa, 2021) as well as mobile phone abuse (Polo et al., 2017; Meldrum et al., 2020). On the other hand, it is found that this kind of dissocial behavior is directly related to violence in all its variants and suicide (Washington, 2015; Vergel et al., 2016; Hinduja and Patchin, 2019; Martín-Baena et al., 2019).

One of the contexts where violent behaviors develop is the academic environment. The study conducted by Xu and Fang (2021) establishes that one of the reasons for these behaviors in the academic environment lies in the degree of achievement in different academic tasks. Such behavior is based on the theory of self-esteem orientation, stating that when subjects cannot obtain a sufficient degree of self-esteem support, they seek other ways to improve their self-esteem (Yiyi et al., 2022). When a person does not achieve the expected academic performance, a decrease in self-esteem is carried out, increasing self-esteem levels through violent behavior toward those who obtain better grades (Xiong et al., 2020).

Furthermore, these behaviors have a negative impact on both physical and mental health (Reisig and Golladay, 2019; Choe et al., 2021). In this situation, emotions play a key role in the appearance and development of violent behaviors (MacCann et al., 2020; Ubago-Jiménez et al., 2021) since they are a checkpoint for controlling one’s own impulses and the self-regulation of the person over himself (Bacon et al., 2014; Beltrán-Catalán et al., 2018; Sorina et al., 2019).

Regarding the importance of violent behaviors at the university setting, and given that little has been done on the interplay between emotional intelligence, self-concept, and academic performance as predictors of violent behaviors, the present study aims to identify and establish the relationship between academic performance, self-concept and violent behavior as a function of academic record average among Spanish university students, by (a) developing an explanatory model of violent behavior and its relationship with self-concept and emotional intelligence and (b) contrasting the structural equation model using a multi-group analysis according to academic record average.

## Materials and methods

### Sample and design

A cross-sectional design was used in order to data collect at one specific time. A non-probabilistic convenience sample was used, in order words, a convenience selection of undergraduate students from the University of Granada, Spain, is used for sample selection. There were 932 university students, 31.7% (*n* = 295) male and 68.3% (*n* = 637) female, with a median age *M* = 20.55; *SD* = 3.673.

#### Instruments

##### Sociodemographic questionnaire

An *ad-hoc* questionnaire was carried out to collect data on gender (male or female), age and average mark (5–6.9 pass, 7–8.9 notable and 9–10 outstanding).

##### Self-concept

In order to determine self-concept, the questionnaire “Self-Concept Form-5 (SF-5),” by García and Musitu (1999) has been used. This scale evaluates the following dimensions: Academic Self-Concept (AS), Social Self-Concept (SS), Emotional Self-Concept (ES), Family Self-Concept (FS) and Physical Self-Concept (PS). It is composed of 30 items answered by means of a 5-choice Likert scale, where 1 = never and 5 = always. For the present research a reliability of *α* = 0.820 was obtained, while for each dimension AS: *α* = 0.767; SS: *α* = 0.698; ES: *α* = 0.749; FS: *α* = 0.754; PS: *α* = 0.744.

##### Emotional intelligence

In the present research, the reduced version of the “Trait Meta-Mood Scale” questionnaire by Fernández-Berrocal et al. (2004) was used. It is composed of 24 items that are answered by means of a 5-point Likert scale, ranging from 1 = Not at all agree to 5 = Strongly agree. This scale also measures the dimensions, differentiating the values for men and women: Emotional Attention (EA), Emotional Clarity (EC) and Emotional Repair (ER). The reliability reported an *α* = 0.901 for this study, and for the EA category *α* = 0.887, EC *α* = 0.915, and ER *α* = 0.871.

##### Violent behavior

The Spanish version of the Little et al. (2003) scale was used to measure violent behaviour (Estévez, 2005). This questionnaire is divided into two categories: Direct Aggression or Indirect Aggression. These categories are subdivided, in turn, into three subscales: Pure, Reactive and Instrumental aggression. It is also answered by means of a 25-item Likert-type scale ranging from 1 = never to 4 = always. Once the scale has been completed, two types of violent behavior are obtained: Direct Aggression, which is generated in a personal encounter between aggressor and victim; and Indirect Aggression, which is considered when aggressors remain anonymous.

#### Procedure

The research process comprised different phases. During the first one, authorization was requested from Department of Corporal Expression and Ethics Committee of the Faculty of Education Sciences of University of Granada (Spain) with the code 1478/CEIH/2020. In the next phase, a document explaining research and study aims was created, and informed consent was requested from participants. Following agreement to participate from 1,025 undergraduates, the instrument was administered individually. Due to the COVID-19 pandemic restrictions, data were collected using the online questionnaire *via* Google Forms. The approximate response time for each subject was 10 min.

During the last phase, the answers of 1,025 undergraduates were checked and 93 questionnaires had to be eliminated because they were not properly fulfilled. Data analysis was carried out between July and September 2021, treating and ensuring participants’ confidentiality. Processing and data analysis were carried out following human research guidelines of the Ethics Committee of University of Granada, and the ethical principles established by Declaration of Helsinki in 1975 and its update in Brazil in 2013.

### Data analysis

The descriptive data analysis was carried out using the statistical programme IBM SPSS Statics 25.0 (IBM Corp, Armonk, NY, USA). Frequency and mean analysis were performed and Cronbach’s alpha was used to determine the internal consistency of the instruments, establishing the reliability index at 95%.

**Figure 1:**
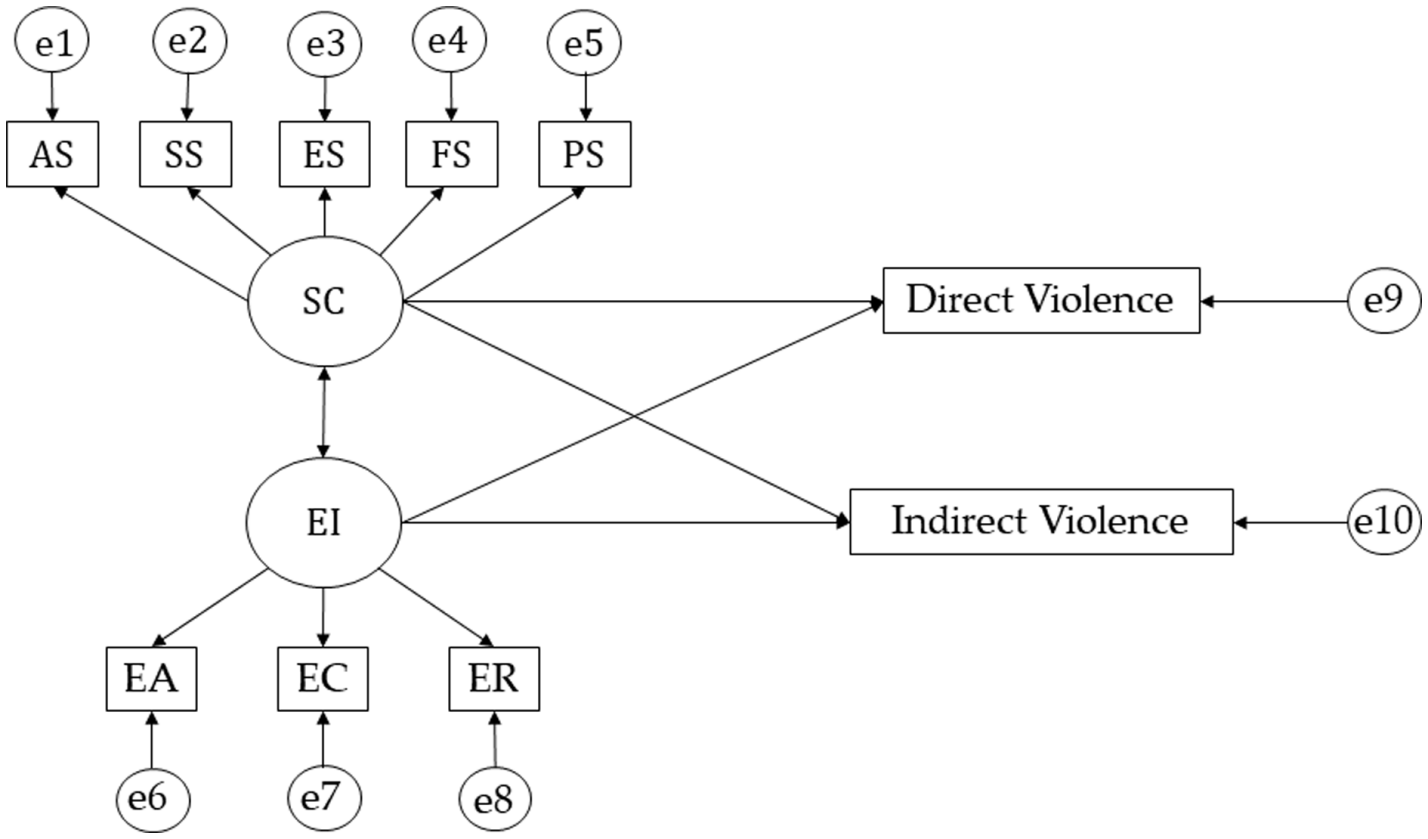
Theoretical model. Self-concept (SC); Academic Self-concept (AS); Social Self-concept (SS); Emotional Self-concept (ES); Family Self-concept (FS); Physical Self-concept (PS); Emotional Intelligence (EI); Emotional Attention (EA); Emotional Clarity (EC); Emotional Repair (ER).

For the structural equation models, the IBM SPSS Amos 26.0 programme (IBM Corp., Armonk, NY, USA) was used to establish the relationships between the variables that make up the theoretical model (Figure 1). Three models have been developed according to the mean academic performance mark obtained (pass, notable and outstanding). The models developed are composed of 10 endogenous variables and two exogenous variables, where causal explanations have been made taking into account the associations obtained between the indicators of measurement reliability. Thus, the measurement error of these variables was also included in the model, which could be controlled and interpreted as multivariate regression coefficients. The one-way arrows represent influence lines between the latent variables and are interpreted from the regression weights and significance level was set at 0.05 using Pearson’s Chi-Square test.

Finally, the model fit was checked after estimating its parameters. Following Kline (2016) and Loehlin and Beaujean (2017), the goodness of fit should be assessed on the Chi-square, whose associated value of *p*s and non-significant values indicate a good model fit. The comparative fit index (CFI) should obtain values above 0.95, the goodness of fit index (GFI) should obtain values above 0.90, the incremental reliability index (IFI) should reflect values above 0.90 and finally, for the root mean square approximation (RMSEA) values below 0.1 indicate an acceptable model fit.

## Results

In the model developed through variables assessed for the sample obtaining an average of pass marks (5–6.99), a good fit has been shown for the different indices. The Chi-square analysis showed a significant value of *p* (*X*^2^ = 297.755; df = 32; pl = 0.000), but these data cannot be interpreted independently due to the influence of susceptibility and sample size (Tenenbaum and Eklund, 2007), so other standardized fit indices have been used. The comparative fit index (CFI) analysis obtained a value of 0.933, the normalized fit index (NFI) analysis obtained a value of 0.905, the incremental fit index (IFI) was 0.961 and the Tucker-Lewis index (TLI) obtained a value of 0.922, all of which were excellent. In addition, the root mean square error of approximation analysis (RMSEA) obtained a value of 0.032.

**Figure 2:**
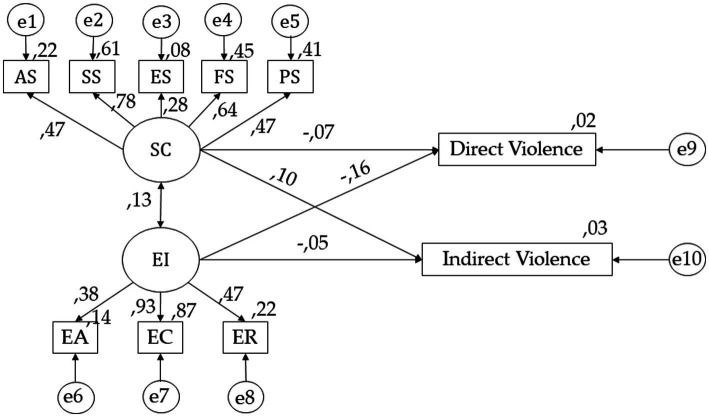
SEM for undergraduates with a pass average mark. Self-concept (SC); Academic Self-concept (AS); Social Self-concept (SS); Emotional Self-concept (ES); Family Self-concept (FS); Physical Self-concept (PS); Emotional Intelligence (EI); Emotional Attention (EA); Emotional Clarity (EC); Emotional Repair (ER).

Figure 2 and Table 1 show the regression weights of the theoretical model, with statistically significant differences at *p* < 0.05 and *p* < 0.001. In relation to self-concept, positive relationships are observed with AS (*r* = 0.470), SS (*p* < 0.001; *r* = 0.781), FS (*p* < 0.001; *r* = 0.668), PS (*p* < 0.001; *r* = 0.471) and ES (*p* < 0.001; *r* = 0.276). Continuing with emotional intelligence, positive relationships are observed with ER (*r* = 0.474), EC (*p* < 0.001; *r* = 0.931) and EA (*p* < 0.001; *r* = 0.375). Looking at direct violence, it is observed that it shows a negative relationship with self-concept (*r* = −0.066) and emotional intelligence (*r* = −0.159). Finally, looking at indirect violence, positive relationships are observed with self-concept (*p* < 0.05; *r* = −0.096) and negative relationships with emotional intelligence (*r* = −0.051).

**Table 1:** Structural model of the theoretical model for pass average mark.

Associations between variables	R.W.				S.R.W.
	Estimations	E.E.	C.R.	*p*	Estimations
AS ← SC	1.000				0.470
SS ← SC	1.275	0.235	5.423	***	0.781
FS ← SC	1.007	0.187	5.385	***	0.668
PS ← SC	1.130	0.248	4.554	***	0.471
ES ← SC	0.707	0.228	3.101	***	0.276
ER ← EI	1.000				0.474
EC ← EI	2.076	0.674	3.081	***	0.931
EA ← EI	0.818	0.190	4.300	***	0.375
EI ↔ SC	0.014	0.011	1.335	**	0.130
Direct Violence ← SC	−0.050	0.062	−0.811	0.417	−0.066
Indirect Violence ← SC	0.060	0.076	−1.891	**	0.096
Direct Violence ← EI	−0.143	0.047	−1.262	0.207	−0.159
Indirect Violence ← EI	−0.037	0.055	−0.671	0.502	−0.051

Regression Weights (R.W); Standardized Regression Weights (S.R.W); Estimation Error (E.E); Critical Relation (C.R). Self-Concept (SC); Academic Self-Concept (AS); Social Self-Concept (SS); Emotional Self-Concept (ES); Family Self-Concept (FS); Physical Self-Concept (Ps); Emotional. Intelligence (EI); Emotional Attention (EA); Emotional Clarutyl (EC); Emotional Reapir (ER). ***p < 0,001; **p < 0.05.

Furthermore, the model developed for students with an average of notable showed good scores for each of the different indices. The Chi-square analysis showed a significant value of *p* (*X*^2^ = 231.490; df = 32; pl = 0.000). The comparative fit index (CFI) analysis obtained a value of 0.945, the normalized fit index (NFI) analysis obtained a value of 0.920, the incremental fit index (IFI) was 0.941 and the Tucker-Lewis index (TLI) obtained a value of 0.909, all of which were excellent. In addition, the root mean square error of approximation analysis (RMSEA) obtained a value of 0.039.

**Figure 3:**
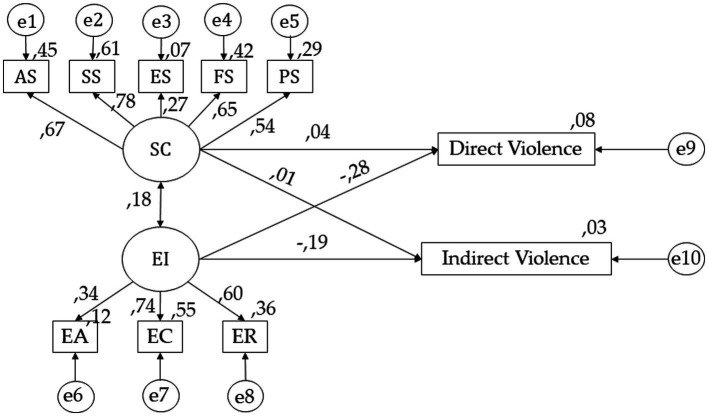
SEM for undergraduates with a notable average mark. Self-concept (SC); Academic Self-concept (AS); Social Self-concept (SS); Emotional Self-concept (ES); Family Self-concept (FS); Physical Self-concept (PS); Emotional Intelligence (EI); Emotional Attention (EA); Emotional Clarity (EC); Emotional Repair (ER).

In Figure 3 and Table 2, positive relationships with AS (*r* = 0.069), SS (*p* < 0.001; *r* = 0.779), FS (*p* < 0.001; *r* = 0.646), PS (*p* < 0.001; *r* = 0.538) and ES (*p* < 0.001; *r* = 0.271) are observed. Following emotional intelligence, positive relationships were found with ER (*r* = 0.603), EC (*p* < 0.001; *r* = 0.739), EA (*p* < 0.001; *r* = 0.341) and self-concept (*p* < 0.05; *r* = 0.184). For the direct violence, positive relationships are observed with self-concept (*r* = 0.041) and emotional intelligence (*r* = 0.010). Finally, for indirect violence, negative relationships were found with self-concept (*p* < 0.001; *r* = −0.282) and emotional intelligence (*p* < 0.001; *r* = −0.187).

**Table 2:** Structural model of the theoretical model for notable average mark.

Associations between variables	R.W.				S.R.W.
	Estimations	E.E.	C.R.	*p*	Estimations
AS ←SC	1.000				0.669
SS ← SC	0.896	0.067	13.363	***	0.779
FS ← SC	0.727	0.059	12.411	***	0.646
PS ← SC	0.886	0.082	10.757	***	0.538
ES ← SC	0.476	0.083	5.762	***	0.271
ER ← EI	1.000				0.603
EC ← EI	1.189	0.165	7.194	***	0.739
EA ← EI	0.510	0.082	6.190	***	0.341
EII ↔ SC	0.039	0.013	3.039	**	0.184
Direct Violence ← SC	0.021	0.024	0.866	0.386	0.041
Indirect Violence ← SC	0.006	0.030	−3.542	0.832	0.010
Direct Violence ← EI	−0.132	0.026	−5.136	***	−0.282
Indirect Violence ← EI	−0.107	0.030	0.213	***	−0.187

Regression Weights (R.W); Standardized Regression Weights (S.R.W); Estimation Error (E.E); Critical Relation (C.R). Self-Concept (SC); Academic Self-Concept (AS); Social Self-Concept (SS); Emotional Self-Concept (ES); Family Self-Concept (FS); Physical Self-Concept (Ps); Emotional Intelligence (EI); Emotional Attention (EA); Emotional Clarutyl (EC); Emotional Reapir (ER). ***p < 0.001; ** *p* < 0.05.

The last model developed showed good scores for each of the different indices. The Chi-square analysis showed a significant value of *p* (*X*^2^ = 229.431; df = 32; pl = 0.000). The comparative fit index (CFI) analysis obtained a value of 0.963, the normalized fit index (NFI) analysis obtained a value of 0.919, the incremental fit index (IFI) was 0.929 and the Tucker-Lewis index (TLI) obtained a value of 0.907, all of which were excellent. In addition, the root mean square error of approximation analysis (RMSEA) obtained a value of 0.043.

**Figure 4:**
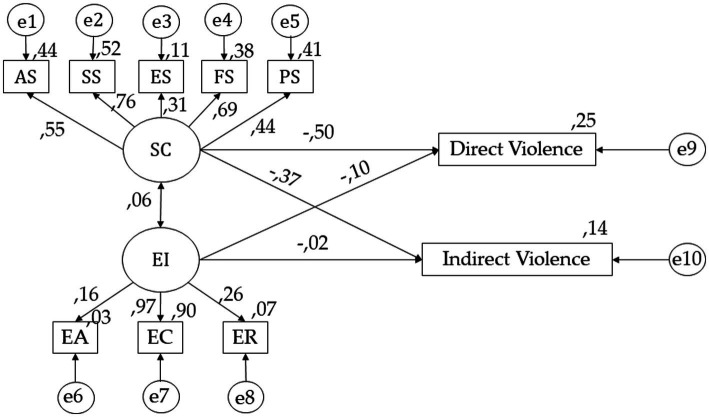
SEM for undergraduates with an a outstanding average mark. Self-concept (SC); Academic Self-concept (AS); Social Self-concept (SS); Emotional Self-concept (ES); Family Self-concept (FS); Physical Self-concept (PS); Emotional Intelligence (EI); Emotional Attention (EA); Emotional Clarity (EC); Emotional Repair (ER).

As shown in Figure 4 and Table 3, for self-concept, positive relationships are observed with AS (*p* < 0.001; *r* = 0.549), SS (*p* < 0.001; *r* = 0.762), FS (*p* < 0.001; *r* = 0.687), PS (*p* < 0.001; *r* = 0.445) and ES (*p* < 0.05; *r* = 0.313). In terms of emotional intelligence, positive relationships were obtained with ER (*r* = 0.264), EC (*r* = 0.901), EA (*p* < 0.05; *r* = 0.160), and self-concept (*p* < 0.001; *r* = 0.057). For direct violence, it shows negative relationships with self-concept (*p* < 0.001; *r* = −0.497) and emotional intelligence (*p* < 0.005; *r* = −0.104). Finally, for indirect violence, a negative relationship is obtained for self-concept (*r* = −0.371) and emotional intelligence (*p* < 0.05; *r* = −0.023).

**Table 3:** Structural model of the theoretical model for outstanding average mark.

Associations between variables	R.W.				S.R.W.
	Estimations	E.E.	C.R.	*p*	Estimations
AS ← SC	1.000				0.549
SS ← SC	0.969	0.188	5.165	***	0.762
FS ← SC	0.790	0.159	4.982	***	0.687
PS ← SC	0.964	0.265	3.772	***	0.445
ES ← SC	0.559	0.197	2.839	**	0.313
ER ← EI	1.000				0.264
EC ← EI	7.991	12.203	0.655	0.513	0.901
EA ← EI	0.503	0.161	3.117	**	0.160
EII ↔ SC	0.004	0.008	0.541	0.589	0.057
Direct Violence ← SC	−0.376	0.092	−4.088	***	−0.497
Indirect Violence ← SC	0.037	0.062	0.596	0.551	−0.371
Direct Violence ← EI	0.151	0.061	2.460	**	−0.104
Indirect Violence ← EI	−0.317	0.097	−3.257	**	−0.023

Regression Weights (R.W); Standardized Regression Weights (S.R.W); Estimation Error (E.E); Critical Relation (C.R). Self-Concept (SC); Academic Self-Concept (AS); Social Self-Concept (SS); Emotional Self-Concept (ES); Family Self-Concept (FS); Physical Self-Concept (Ps); Emotional Intelligence (EI); Emotional Attention (EA); Emotional Clarutyl (EC); Emotional Reapir (ER). ***p < 0.001; **p < 0.05.

## Discussion

This study showed the relationships between academic performance, self-concept, emotional intelligence, and violent behavior in undergraduates. The results obtained answered the aims stated in the research. Likewise, recent studies have been found that present similar characteristics and relate the research variables (Molero et al., 2014; Elipe et al., 2015; García-Martínez et al., 2022).

Based on a first approach to the comparative analysis, the data obtained show relationships between self-concept, emotional intelligence and violent behavior (Baroncelli and Ciucci, 2014; Estévez et al., 2019; Cañas et al., 2020; Skrzypiec et al., 2021). In fact, it can be observed that university students with an average of outstanding marks have a negative relationship between their self-concept and the two kinds of violence. Similar results are found in the meta-analysis of Samara et al. (2021) who analysed studies carried out in children and adolescents. They also found that students with lower school performance are those who tend to be victims of violent behavior. On the other hand, Ma et al. (2009) found that lower academic performance was associated with high levels of disruptive behavior in adolescents. Moreover, numerous studies confirm the relationship between school bullying and lower grades (Román and Murillo, 2011; Sinkkonen et al., 2014; Droppert et al., 2019; Pörhölä et al., 2019). The results showed that undergraduates with a pass average mark are more likely to engage in a type of indirect violence such as cyberbullying (Kowalski and Limber, 2013; Young-Jones et al., 2015; Martínez-Ramón et al., 2019).

The research carried out by Kowalski and Limber (2013) states that people who perform poorly academically tend to show behavior based on physical or virtual bullying toward those who perform better academically. Furthermore, the research carried out by Martínez-Ramón et al. (2019) states that the act of bullying within the university environment is undergoing a process of evolution, as it is moving from physical violence to covert violence, which is based on isolating and underestimating the victim. This type of academic bullying leads to a reduction in basic academic skills, producing a reduction in academic motivation levels and giving rise to an increase in anxiety levels (Young-Jones et al., 2015).

In addition, it is also observed that emotional intelligence correlates negatively with both forms of violence for students with a pass average mark and a notable average mark. Our findings give credence to Elipe et al. (2015) who confirm how students show a better emotional adaptation to different kinds of violence.

However, it has been found that undergraduates with a higher average mark have a negative correlation between their emotional condition and direct and indirect violence. Perhaps this could be caused by a higher stress exposure due to a higher academic performance (Lee, 2017). Also, their desire to have a higher average mark makes students more competitive, which causes them to present emotions such as anger, annoyance or despair (Elipe et al., 2015). The above data support previous investigations that have shown how suffering any type of school violence is associated with low academic performance (Ramírez-Granizo et al., 2019; Torres et al., 2019; Tekel and Karadag, 2020).

According to the above, it is worth highlighting that the present study shows the relationship between self-concept, emotional intelligence, violence behaviors and their impact on academic performance. Therefore, emotional education should be promoted in order to develop self-control in anxiety and stressful situations to prevent violent behavior. It is also suggested that teachers need to increase their teacher-student interpersonal behaviors, by drawing on the tenets of positive psychology and rhetorical/relational goal theory (Xie and Derakhshan, 2021). Likewise, it is also necessary to strengthen and reward efforts to obtain a better academic performance in order to prevent emotional problems and adverse emotions such as anger, boredom, and violence.

### Limitations and future prospectives

This research has some limitations, being its descriptive and cross-sectional design the main limitation, as it was only possible to measure the variables at a single point in time. Taking into account the sample, it does not allow generalizations to be made as it belongs to a single specific area. It is important to highlight the moment when the research was carried out, a period of high incidence of the COVID-19 virus. This limited access to a larger number of participants. It is also important to emphasize the presence of strange variables that may have influenced the variables studied in this research.

In terms of future perspectives, the aim is to design an intervention aimed at emotional regulation and improvement of self-concept, in order to reduce disruptive behavior. In this case, the proposed study would demonstrate a mixed method, offering a quantitative and qualitative aspect. In addition, it would be interesting to know the aggressors’ motives for bullying their peers. Other variables to be studied could also be added, such as socio-economic level and the degree of family functionality.

Recently, a new concept has emerged: emotioncy, which refers to the cognitive link between emotions induced by the senses (Pishghadam et al., 2019, 2022). It would be recommended to consider the concept of emotioncy in further studies.

In general, an acceptable value has been obtained for the different parameters of the general equation. This study shows how the academic performance can play a fundamental role in the appearance of violent behavior. A positive relationship between emotional intelligence and direct and indirect violence is observed for students with an outstanding average mark. In addition, positive relationships are observed between indirect violence and self-concept. Negative relationships are also observed between self-concept and direct violence.

Therefore, it is possible to affirm that emotional intelligence and self-concept have a direct influence on the development of violent behavior, with academic performance playing a key role in the assessment of each variable.

## Data availability statement

The original contributions presented in the study are included in the article/supplementary material, further inquiries can be directed to the corresponding author.

## Ethics statement

The studies involving human participants were reviewed and approved by Department of Corporal Expression and Ethics Committee of the Faculty of Education Sciences of University of Granada (Spain) with the code 1478/CEIH/2020. Written informed consent for participation was not required for this study in accordance with the national legislation and the institutional requirements.

## Author contributions

JU-J, SC-R, JO-M, and EM-I: idea, state of the art, methodology, and data analysis, results, discussion and conclusions, and final revision. JU-J, SC-R, and JO-M: literature review (state of the art). JU-J and EM-I: original draft. All authors contributed to the article and approved the submitted version.

## Conflict of interest

The authors declare that the research was conducted in the absence of any commercial or financial relationships that could be construed as a potential conflict of interest.

## Publisher’s note

All claims expressed in this article are solely those of the authors and do not necessarily represent those of their affiliated organizations, or those of the publisher, the editors and the reviewers. Any product that may be evaluated in this article, or claim that may be made by its manufacturer, is not guaranteed or endorsed by the publisher.
